# Comparison of the effectiveness of low pressure pneumoperitoneum with profound muscle relaxation during laparoscopic donor nephrectomy to optimize the quality of recovery during the early post-operative phase: study protocol for a randomized controlled clinical trial

**DOI:** 10.1186/s13063-015-0887-7

**Published:** 2015-08-12

**Authors:** Denise M. D. Özdemir-van Brunschot, Gert J. Scheffer, Albert Dahan, Janneke E. E. A. Mulder, Simone A. A. Willems, Luuk B. Hilbrands, Frank C. H. d’Ancona, Rogier A. R. T. Donders, Kees J. H. M. van Laarhoven, Michiel C. Warlé

**Affiliations:** Department of Surgery, Division of Vascular and Transplant Surgery, Radboud University Medical Center, Geert Grooteplein-Zuid 10, Nijmegen, 6525 GA The Netherlands; Department of Anesthesiology, Radboud University Medical Center, Nijmegen, The Netherlands; Department of Anesthesiology, Leiden University Medical Center, Leiden, The Netherlands; Department of Nephrology, Radboud University Medical Center, Nijmegen, The Netherlands; Department of Urology, Radboud University Medical Center, Nijmegen, The Netherlands; Department of Health Evidence, Radboud University Medical Center, Nijmegen, The Netherlands

**Keywords:** Deep neuromuscular block, Laparoscopic donor nephrectomy, Laparoscopy, Low pressure, Sugammadex, Randomized controlled trial

## Abstract

**Background:**

Since technique modifications of laparoscopic donor nephrectomy, e.g. retroperitoneoscopic donor nephrectomy or hand-assistance, have not shown significant benefit regarding safety or improvement of recovery, further research should focus on improving postoperative recovery. The use of low pressure pneumoperitoneum has shown to significantly reduce postoperative pain after laparoscopy. To facilitate the use of low pressure pneumoperitoneum, deep neuromuscular block will be used.

**Methods/Design:**

This trial is a phase IV, single center, double-blind, randomized controlled clinical trial in which 64 patients will be randomized to: low pressure pneumoperitoneum (6 mmHg) and deep neuromuscular block or normal pressure pneumoperitoneum (12 mmHg) and deep neuromuscular block. Deep neuromuscular block is defined as post tetanic count < 5. Primary outcome measurement will be Quality of Recovery-40 questionnaire (overall score) on day 1.

**Discussion:**

This study is the first randomized study to assess the combination of low pressure pneumoperitoneum in combination with deep neuromuscular block from a patients’ perspective. The study findings may also be applicable for other laparoscopic procedures.

**Trial registration:**

The trial was registered at trials.gov (NCT02146417) in July 2014.

## Background

Since the introduction of laparoscopic donor nephrectomy in 1995 by Ratner et al. [1], several trials have been performed comparing laparoscopic versus open donor nephrectomy. A Cochrane systematic review has shown that laparoscopic donor nephrectomy is associated with less post-operative pain, better quality of life and shorter hospital stay for the donor [2]. Therefore, in most countries, laparoscopic donor nephrectomy is nowadays the procedure of first choice.

So far, modifications of the technique of laparoscopic donor nephrectomy, i.e. hand-assisted and/or retroperitoneoscopic approaches, did not show a significant benefit with regard to safety as reflected by the conversion to open donor nephrectomy and postoperative complications [3–6]. Therefore, further research should focus on early postoperative recovery. Postoperative recovery is largely determined by the consequences of postoperative pain and its concomitant use of opioids. Measures to reduce postoperative pain will also reduce opioid-associated side-effects, including postoperative nausea and vomitus.

A recent pilot study performed by our group has shown that low pressure pneumoperitoneum (7 mmHg) was feasible and significantly reduced postoperative pain scores during the first 72 hours after surgery [7]. Others have shown that low pressure pneumoperitoneum in other laparoscopic procedures is associated with a reduction of post-operative pain and analgesic consumption [8–10]. However, low pressure pneumoperitoneum was also associated with longer operation time, probably due to less optimal perioperative visibility. To facilitate low pressure pneumoperitoneum deep neuromuscular block can be used; Martini et al. have shown that perioperative visibility can be improved by deep neuromuscular block in normal pressure pneumoperitoneum [11]. Therefore, deep neuromuscular block might become a prerequisite for the use of low pressure pneumoperitoneum.

However, the extended effects of deep neuromuscular block may lead to postoperative complications, including airway obstruction, hypoxia, pneumonia and residual muscle paralysis, necessitating prolonged stay in the post-anaesthesia care unit [12, 13]. To prevent this, sugammadex, a modified γ-cyclodextrine that binds to rocuronium in plasma and tissues, will be administrated.

Our hypothesis is that, for patients undergoing laparoscopic donor nephrectomy, low pressure pneumoperitoneum leads to improved postoperative recovery as compared to patients with standard pressure pneumoperitoneum.

## Methods/Design

The protocol of the study was approved by the local ethics committee (NL48056.091.14, Central Committee on Research involving Human Subjects, Arnhem – Nijmegen) and registered at clinicaltrials.gov (NCT02146417). This single center, double-blind, randomized controlled trial will be performed at the Radboud University Medical Center. Inclusion will be performed by the research physician after written informed consent.

### Study population

A total of 64 patients will be randomized based on a computer-generated list, using sealed, opaque envelopes (1:1) to either low pressure pneumoperitoneum (6 mmHg) and deep neuromuscular block or normal pressure pneumoperitoneum (12 mmHg) and deep neuromuscular block. Stratification for gender and site of donor nephrectomy will be used. As the range in age of live kidney donors in our center is relatively small (between 40 and 60 years), we will not stratify for age. All adult individuals who were considered to be suitable for live kidney donation after multidisciplinary discussion (nephrologist, vascular surgeon and urologist) are eligible for this study.

### Inclusion criteria

- Informed consent obtained
- Age over 18 years

### Exclusion criteria

- Insufficient control of the Dutch language to read the patient information and to fill out the questionnaires
- Chronic use of analgesics or psychotropic drugs
- Use of nonsteroidal anti-inflammatory drugs < 5 days before surgery
- Known or suspect allergy to rocuronium or sugammadex
- Significant liver or renal dysfunction
- Neuromuscular disease
- Pregnant or breastfeeding
- Indication for rapid sequence induction

Liver dysfunction is defined as alanine aminotransferase (ALAT) and/or aspartate aminotransferase (ASAT) more than twice the upper limit. Renal dysfunction is defined as serum creatinine twice the normal level (upper limit of 201 μmol/l) and/or glomerular filtration rate < 60 ml/minute. Both renal and liver dysfunction are extremely rare in live kidney donors.

### Study protocol

Before arrival at the operating room, all monitors and screens displaying information regarding the intra-abdominal pressure will be covered for the anesthesiologist, surgeons, research physician and scrub nurses. An independent scrub nurse from an adjacent operating room consults the randomization tool and will be informed about the allocation of the treatment. Subsequently, the independent scrub nurse installs the pneumoperitoneum insufflation pressure at 6 mmHg or 12 mmHg.

All laparoscopic procedures will be performed by two teams consisting of a fixed combination of one vascular surgeon and one urologist. The surgical rating score will be scored by the primary surgeon. Each surgeon has performed more than 30 laparoscopic donor nephrectomies.

Anaesthesia will be induced with propofol 1–3 mg/kg and sufentanyl 0.2–0.5 μg/kg. Deep neuromuscular block will be induced by rocuronium bolus 1.0 mg/kg. To calibrate the train-of-four (TOF)-Watch (TOF-Watch SX, MSD, Haarlem, the Netherlands), first a tetanic ulnar nerve stimulus (50 Hz for 5 seconds) will be administered. Thereafter, the TOF-Watch will be calibrated, followed by 3 measurements to ensure that the TOF ratio differs by less than 5 %. If the TOF ratio differs > 5 % the TOF-Watch will be recalibrated. Anesthesia will be maintained by sufentanyl 0.05–0.5 μg/kg/h and sevoflurane. Deep neuromuscular block will be maintained by a continuous infusion of rocuronium (0.3 mg/kg/h). In case of persistent post-tetanic count (PTC) values of 0, continuous infusion will be paused; when PTC increases to > 5, the pump speed will be increased and/or an extra bolus of rocuronium will be given.

**Fig. 1:**
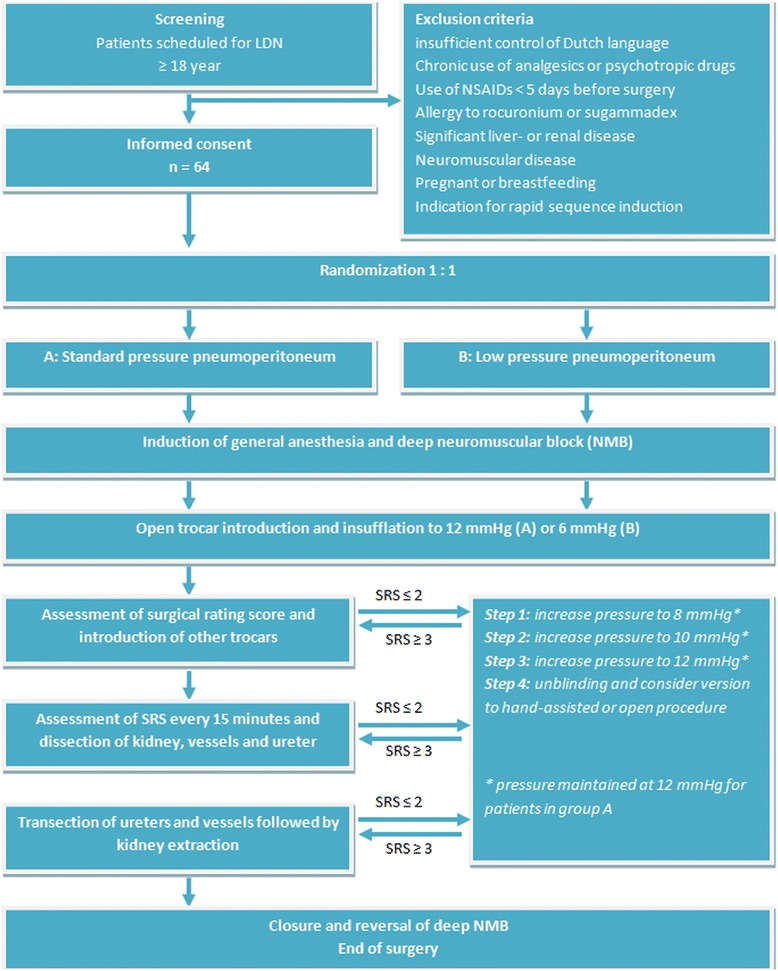
Flow chart

After open introduction of the first trocar, the surgeon assesses surgical rating scale (SRS) according to Martini et al. (see Table 1) [11]. The SRS score will only be based on actual intra-abdominal conditions, e.g. in case of problems with the laparoscopic camera, this will be excluded as cause of poor visibility. If SRS is ≥ 3 the procedure will be continued. If the SRS is ≤ 2 the pneumoperitoneum pressure will be increased stepwise (by the independent scrub nurse), according to Fig. 1. Before each step (introduction of trocars, dissection of the kidney and kidney extraction), surgical conditions are assessed by the surgeon. During the dissection phase surgical conditions will be assessed every 15 minutes. In case increasing the intra-abdominal pressure to normal (12 mmHg) does not improve the SRS to ≥ 3, the surgeon decides to take further action, e.g. conversion to hand-assisted or open donor nephrectomy, but will not be informed about the initial pressure. The (independent) research physician will register intra-operative parameters (e.g. blood loss, first warm ischemia time, conversion to open or hand-assisted donor nephrectomy and intra-operative complications).

**Table 1:** Assessment of surgical space condition, according to Martini et al. [11]

Scale		Description
1	Extremely poor conditions	The surgeon is unable to work because of coughing or because of the inability to obtain a visible laparoscopic field because of inadequate muscle relaxation. Additional neuromuscular blocking agents must be given
2	Poor conditions	There is a visible laparoscopic field, but the surgeon is severely hampered by inadequate muscle relaxation with continuous muscle contractions, movements, or both with the hazard of tissue damage. Additional neuromuscular blocking agents must be given
3	Acceptable conditions	There is a wide visible laparoscopic field but muscle contractions, movements, or both occur regularly causing some interference with the surgeon’s work. There is the need for additional neuromuscular blocking agents to prevent deterioration
4	Good conditions	There is a wide laparoscopic field with sporadic muscle contractions, movements, or both. There is no immediate need for additional neuromuscular blocking agents unless there is the fear of deterioration
5	Optimal conditions	There is a wide visible laparoscopic working field without any movement or contractions. There is no need for additional neuromuscular blocking agents

Pain management is achieved by patient-controlled analgesia with Dipidolor (piritramide) (bolus 1 mg, lock-out 6 minutes) and acetaminophen (total 4000 mg daily). When no complications occur and pain management is satisfactory: on day 2, patient-controlled analgesia will be stopped and replaced by oral analgesics. On day 0, post-operative pain will be assessed every 4 hours; thereafter, pain will be assessed every 8 hours. On day 0, patients are offered a liquid meal; thereafter, patients are encouraged to eat regular meals. The research physician will assess all post-operative pain scores and perform daily evaluation with regard to the use of analgesics and anti-emetics and urine output. In case of nausea and/or vomiting, ondansetron (4 mg intravenous, maximum 12 mg) is given; second choice is metoclopramide (10 mg intravenous, maximum 30 mg). Since the spouse or child of the patient is frequently the recipient of the donor, patients are often admitted for longer than strictly medically necessary. Therefore, the following discharge criteria will be evaluated daily: 1) satisfactory pain management with oral analgesia, 2) passage of flatulence and feces, 3) ability to walk over the ward, 4) ability to wash and change clothes independently.

### Outcome measures

Primary outcome measure is total score of the Quality of Recovery-40 (QoR-40) questionnaire on post-operative day 1. The QoR-40 questionnaire provides a global score and sub-score in 5 dimensions: patient support, comfort, emotions, physical independence and pain. An improvement in the quality of recovery is directly related to patient satisfaction [14]. Also, there is a relationship between quality of recovery in the days and weeks after surgery, with quality of life up to 3 years after cardiac surgery [15]. In accordance with the literature, we expect the greatest difference of low versus standard pressure pneumoperitoneum regarding the QoR-40 at post-operative day 1 [16–18].

Secondary outcome measures include:

- Intra-operative parameters (e.g. intra-operative complications, conversion to open or hand-assisted donor nephrectomy, duration of pneumoperitoneum, estimated blood loss, first warm ischemia time)
- Post-operative complications
- Use of anti-emetics and analgesics
- Post-operative pain
- Nausea and vomiting
- Discharge criteria
- Return to work
- Serum creatinine

The QoR-40 questionnaire is assessed on days −1, 1, 2 and 3 and week 1. Components of pain are assessed on days −1, 0, 1, 2 and 3, week 6 and month 3. On the day of surgery, components of pain are measured 1 to 2 hours after surgery. Return to work questionnaire is measured at weeks 4 and 6 and month 3. Medication use is, during hospital admission, evaluated daily. Serum creatinine is measured on days −1 and 2 and after 6 weeks. Other laboratory investigations are only performed when indicated by the attending physician. For a complete overview of the time schedule see Table 2.

**Table 2:** Time schedule

	D −1	D 0	D 1	D 2	D 3	W 1	W 4	W 6
Questionnaires								
QoR-40	X		X	X	X	X		
Return to work							X	X
Medication use								
Use of opioids	X	X	X	X	X			X
Use of other analgesics	X	X	X	X	X			X
Evaluation of anti-emetic use	X	X	X	X	X			X
Clinical parameters								
Components of pain	X	X	X	X	X			X
Nausea score	X	X	X	X	X			
Surgical parameters		X						
Urine output		X	X	X	X			
Evaluation of complications		X	X	X	X			X
Discharge criteria			X	X	X			
Laboratory values								
Serum creatinine	X			X				X

*D* day, *W* week

### Ethics, informed consent

The Central Committee on Research involving Human Subjects, Arnhem-Nijmegen, approved the protocol. Oral and written informed consent will be obtained from the patient before inclusion.

### Adverse events and reactions

Our pilot study has shown that the use of low pressure pneumoperitoneum in laparoscopic donor nephrectomy is feasible and can decrease post-operative pain [7]. A Cochrane review comparing low pressure pneumoperitoneum in laparoscopic cholecystectomy [8] showed no difference in mortality, morbidity or conversion to open cholecystectomy between both groups.

Martini et al. have shown that deep neuromuscular block increases surgical visibility during laparoscopy with normal intra-abdominal pressure [11]. Deep neuromuscular block can be achieved with rocuronium. Rocuronium can safely be administrated to patients with cardiac and/or pulmonary comorbidity. To monitor neuromuscular blockade, TOF measurements will be assessed every 10 minutes during the procedure. To avoid residual paralysis, necessitating prolonged stay on the post-anesthesia care unit, sugammadex will be administered. Patients will only be extubated when TOF is > 90 %. Furthermore, patients will stay at the post-anesthesia care unit for 2 hours, to ensure adequate neuromuscular function.

### Sample size calculation

A sample size of 32 patients per group is needed to provide 80 % power to detect a 10-point difference in the overall score in the QoR-40 scale at post-operative day 1. A 10-point difference represents a minimal clinically relevant difference in the QoR-40 scale [19–22]. The QoR-40 score after laparoscopic donor nephrectomy is not previously assessed. For other procedures, standard deviation of QoR-40 scores varies between 12 and 23 points [15, 23–27] with one study investigating the QoR-40 score after laparoscopic surgery [18]. Data from this study indicate a standard deviation of 14 in patients after laparoscopic hysterectomy. As the group of living kidney donors is highly homogeneous, we do not expect a higher variation in the quality of recovery as compared to patients after laparoscopic hysterectomy. Therefore, we used a standard deviation of 14 for the sample size calculation. In total, 64 patients are needed for the proposed trial.

### Statistical analysis

The data analysis will be based on an intention-to-treat approach. Since we stratify for gender and side of donor nephrectomy, factorial analysis of variance (ANOVA) with a custom design will be used for statistical analysis. *P* values < 0.05 will be considered statistically significant. Statistical analyses will be performed with SPSS 22.0 (SPSS Inc., Chicago, IL, USA). A *P* value of 0.05 is considered statistically significant.

## Discussion

Our primary hypothesis is that the use of low pressure pneumoperitoneum, facilitated by deep neuromuscular block, improves the quality of recovery during the early post-operative phase after laparoscopic donor nephrectomy.

Low pressure pneumoperitoneum may compromise visibility of the surgical field. In this trial, safety of low pressure pneumoperitoneum is ensured by assessment of the SRS before each important step and every 15 minutes during the dissection phase. In case of insufficient progression or visibility, intra-abdominal pressure will be increased step-wise to standard pressure if needed. Martini et al*.* have compared the relationship between level of neuromuscular block and SRS in laparoscopic surgery [11]. In this trial normal pressure was used in all patients and deep neuromuscular block was associated with better surgical visibility. Staehr-Rye et al. recently completed a trial comparing surgical space conditions in either deep muscle relaxation or moderate block during low-pressure laparoscopic cholecystectomy [28]. Optimal surgical space conditions were observed in 7 of 25 patients allocated to deep neuromuscular block versus 1 of 23 patients allocated to moderate block.

The main strength of this study is that we investigate the clinical benefit of the use of low pressure pneumoperitoneum in a complex laparoscopic procedure. Also, this is the first study in which the effect of low pressure pneumoperitoneum is investigated in combination with deep neuromuscular block from a patients’ perspective. As standard quality of life questionnaires are not designed to measure quality of recovery of surgery, we use the validated QoR-40 questionnaire. A systematic review performed in 2012 has shown that the QoR-40 questionnaire is a suitable assessment of post-operative quality of recovery in a range of clinical and research situations [29]. One of the limitations is the fact that the surgeon cannot be completely blinded. A flaccid abdominal wall may indicate the use of low intra-abdominal pressure. To assess the efficacy of our blinding procedure, the surgeon will be asked at the end of the procedure whether low or standard pressure was used. Another important limitation is that there is not a validated instrument for measuring perioperative conditions. In the trial by Martini et al., eight surgeons, specialized in laparoscopic surgery, independently scored SRS in the videos [11]. An average κ statistic of 0.50 was found, indicating moderate agreement. Currently, a validation study of subjective rating scales to assess surgical conditions in laparoscopic surgery is being performed (NCT02079337).

If this study shows that low pressure pneumoperitoneum in combination with deep neuromuscular block improves the quality of recovery after laparoscopic donor nephrectomy, these results may be translated to other complex laparoscopic procedures (e.g*.* laparoscopic upper gastrointestinal and colonic surgery).

## Trial status

Recruiting patients

## Abbreviations

ALAT: alanine aminotransferase
ANOVA: analysis of variance
ASAT: aspartate aminotransferase
QoR-40 questionnaire: Quality of Recovery-40 questionnaire
SRS: surgical rating scale
TOF: train-of-four
